# Genetic signatures of a demographic collapse in a large-bodied forest dwelling primate (*Mandrillus leucophaeus*)

**DOI:** 10.1002/ece3.98

**Published:** 2012-03

**Authors:** Nelson Ting, Christos Astaras, Gail Hearn, Shaya Honarvar, Joel Corush, Andrew S Burrell, Naomi Phillips, Bethan J Morgan, Elizabeth L Gadsby, Ryan Raaum, Christian Roos

**Affiliations:** 1 Department of Anthropology, University of Oregon,308 Condon Hall, Eugene, Oregon 97403.; 2 Wildlife Conservation Research Unit, University of Oxford,Abingdon Road, Tubney, Oxfordshire, OX13 5QL, United Kingdom.; 3 Department of Biology and Bioko Biodiversity Protection Program, Drexel University,3141 Chestnut Street, Philadelphia, Pennsylvania 19104.; 4 Department of Anthropology, New York University,25 Waverly Place, New York, New York 10003.; 5 Department of Biology, Arcadia University,450 South Easton Road, Glenside, Pennsylvania 19038-3295.; 6 San Diego Zoo Global Institute for Conservation Research,15600 San Pasqual Valley Road, Escondido, California 92027.; 7 Department of Psychology, University of Stirling,FK9 4LA, Stirling, Scotland, United Kingdom.; 8 Pandrillus, H.E.P.O.,Box 826, Calabar, Nigeria.; 9 Department of Anthropology, Lehman College and City University of New York Graduate Center,250 Bedford Park Blvd., West Bronx, New York 10468.; 10 Gene Bank of Primates and Primate Genetics Laboratory, German Primate Center,Kellnerweg 4, 37077 Göttingen, Germany.

**Keywords:** Bayesian Skyline Plot, bottleneck, climate change, Cross-Sanaga-Bioko forests, drill, *Mandrillus*

## Abstract

It is difficult to predict how current climate change will affect wildlife species adapted to a tropical rainforest environment. Understanding how population dynamics fluctuated in such species throughout periods of past climatic change can provide insight into this issue. The drill (*Mandrillus leucophaeus*) is a large-bodied rainforest adapted mammal found in West Central Africa. In the middle of this endangered monkey's geographic range is Lake Barombi Mbo, which has a well-documented palynological record of environmental change that dates to the Late Pleistocene. We used a Bayesian coalescent-based framework to analyze 2,076 base pairs of mitochondrial DNA across wild drill populations to infer past changes in female effective population size since the Late Pleistocene. Our results suggest that the drill underwent a nearly 15-fold demographic collapse in female effective population size that was most prominent during the Mid Holocene (approximately 3-5 Ka). This time period coincides with a period of increased dryness and seasonality across Africa and a dramatic reduction in forest coverage at Lake Barombi Mbo. We believe that these changes in climate and forest coverage were the driving forces behind the drill population decline. Furthermore, the warm temperatures and increased aridity of the Mid Holocene are potentially analogous to current and future conditions faced by many tropical rainforest communities. In order to prevent future declines in population size in rainforest-adapted species such as the drill, large tracts of forest should be protected to both preserve habitat and prevent forest loss through aridification.

## Introduction

How a species has responded to climatic events not only informs us about the past but also offers insight into how the species might cope with future climate change (Davis et al. 2005; Prost et al. 2010). The period during and since the Last Glacial Maximum (LGM) offers an ideal model for investigating this issue. The LGM, which occurred near the end of the Pleistocene at 26.5 Ka (Clark et al. 2009), was a dramatic period of glacial advance and global cooling and was followed by a period of warm and humid climate that lasted through the Early Holocene. Afterwards, fluctuations in aridity in the Mid and Late Holocene (along with human activity) shaped many of the Earth's modern ecosystems (Maley 1996). Recently developed model-based analyses of molecular data allow for the accurate inference of the timing and magnitude of past changes in the effective population size (*N*_e_) of species through this time period (e.g., Beaumont 1999; Drummond et al. 2005). The vast majority of studies that have performed these estimates have focused on taxa of either subtropical (Li et al. 2009; Liao et al. 2010), temperate (Depraz et al. 2008; Gratton et al. 2008; Lessa et al. 2010), alpine (Galbreath et al. 2009), arctic (Shapiro et al. 2004; Campos et al. 2010; Prost et al. 2010), or northern marine habitats (Larmuseau et al. 2009; Canino et al. 2010; Faurby et al. 2010; Marko et al. 2010). Very few studies have used model-based methods to investigate past changes in N_e_ associated with climate change in tropical species, particularly in relation to forest-adapted animals. This lack of knowledge is worrisome given the biodiversity present in tropical forest ecosystems and the dramatic climatic and environmental changes that they are forecasted to undergo in the near future (the next 40–70 years; Beaumont et al. 2011; Heubes et al. 2011). If we are to predict how climate change will affect species in forested tropical regions, we should have a better understanding of how they reacted to environmental changes during and since the Late Pleistocene.

**Figure 1:**
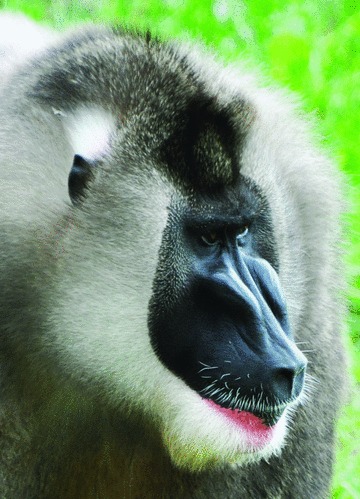
*Mandrillus leucophaeus* male.

In an effort to address this knowledge gap, we investigated the past demography of a large-bodied terrestrial mammal adapted to a rainforest environment–the drill (*Mandrillus leucophaeus*). The drill is an African Old World monkey (family Cercopithecidae) that is endangered, suffers from habitat loss and poaching, and ranks among the highest priorities for African primate conservation (Oates 1996). Like its sole living congenerer (the mandrill; *M. sphinx*), it is thought to have a relatively large home range and form multimale social groups of up to 400+ individuals with females remaining in their natal group (Wild et al. 2005; Astaras 2009). Both species are also highly sexually dimorphic with adult males weighing over three times as much as females and possessing large canines and strikingly colorful perineal hair and skin (red, blue, violet) (Setchell et al. 2001). However, in contrast to the bright red and blue facial skin of male mandrills, male drills have a jet black face with prominent cheek flanges and paranasal ridges framed by a rim of white skin and a red strip below the lower lip (Fig. 1). Also unlike mandrills, drills are primarily limited to a forested environment and are not known to exploit savanna-gallery forest mosaic ecosystems (Astaras 2009).

**Figure 2:**
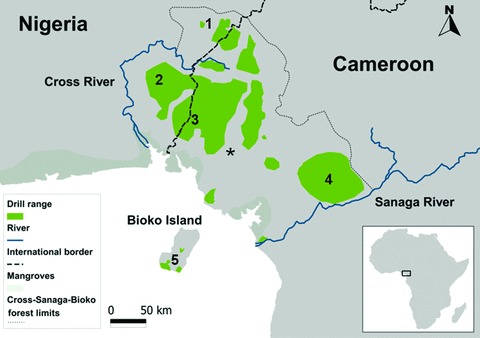
Geographic distribution of the drill (*Mandrillus leucophaeus*) in the Cross-Sanaga-Bioko Coastal forests in West Central Africa (modified from Astaras 2009). Sampled localities are 1 = northern Cross River (several forests in the Boki region, including Afi Mountain and Okwangwo), 2 = southern Cross River (Western Oban Hills), 3 = Korup National Park, 4 = Ebo Forest, 5 = Bioko Island, Asterisk = Lake Barombi Mbo.

**Figure 3:**
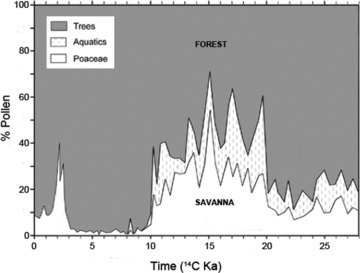
Fossil pollen profile for Lake Barombi Mbo displaying changes in forest coverage since the Late Pleistocene in the geographic range of *Mandrillus leucophaeus* (modified from Bonnefille 2007). Present day is on the left on the *x* -axis. Forest pollens decrease and savanna pollens increase during the Last Glacial Maximum (LGM) and during an arid period in the Mid-Late Holocene. Pollens from aquatic vegetation also rise during cold and/or dry periods as lake levels drop.

The drill is endemic to the Cross-Sanaga-Bioko coastal forests of West Central Africa, which—as their name suggests—extend between the Cross River in Nigeria and the Sanaga River in Cameroon, and on Bioko Island (Equatorial Guinea) (Fig. 2). In the middle of the drill geographic range is Lake Barombi Mbo, which has one of the most complete palynological records in the world since the Late Pleistocene for a forested region (Maley 1996; Maley and Brenac 1998; Bonnefille 2007). Fossil pollen cores from this lake show a severe reduction in rainforest coverage between 24 and 11 Ka, which coincides with the LGM. This is followed by a dramatic forest expansion between 11 and 3 Ka when global temperatures, humidity, and percent forest cover were higher than current levels. At 3 Ka, the forest underwent another dramatic reduction due to drier climate and increased seasonality. Between 2 and 1 Ka the climate becomes more humid, which allows the forest to re-expand (Fig. 3; Bonnefille 2007).

Because it is restricted to a tropical rainforest habitat and exists in a region that has a documented record of environmental change during and since the LGM, the drill offers a rare opportunity to investigate how climate change might have affected a large-bodied tropical forest dwelling mammal. We hypothesize that the species underwent population size changes that mirror the changes in forest coverage in this region (based on the Barombi Mbo pollen profile); increasing and decreasing in population size as forests expanded and receded, respectively. In order to test this hypothesis, we sequenced ∼2,000 base pairs of mitochondrial DNA from drills across the species’ range and used a Bayesian Skyline Plot (BSP) to infer the timing and magnitude of past changes in drill female effective population size (*N*_ef_).

## Materials and Methods

### Sampling strategy

We sampled 54 drills from Nigeria (northern and southern Cross River), Cameroon (Ebo Forest, Korup National Park [KNP]), and Equatorial Guinea (Bioko Island) between the years of 2005–2010 (see Fig. 2 and Table S1). The Nigerian localities included forests in the Boki region (e.g., Afi Mountain, Okwangwo) in the North and Western Oban Hills in the South. All together, the sampled localities encompass the far eastern, western, northern, and southern limits of the drill range, thus capturing the full extent of geographic variation in the species. Subpopulation sampling was also maximized to avoid incorrect inferences of population size change caused by recent immigrants from unsampled populations (Chikhi et al. 2010; Peter et al. 2010).

All Nigerian and one of the Bioko samples were from wild caught captive individuals of known origin that are kept at the Pandrillus colony, while other samples were field collected. The vast majority of biomaterials were of fecal origin, although tissue biopsies from fingertips or liver were collected from some fresh carcasses during surveys of bushmeat offtake in the Malabo (Bioko) market in 2007. We specifically designed the sampling methods so they would not encourage illegal hunting (i.e., no money was exchanged for market samples and groups were not habituated in the wild).

### Genetic loci

We analyzed two loci (“Brown” region and Cytochrome b) that together encompass 2,076 base pairs of the mitochondrial genome. Mitochondrial DNA was chosen because it has a relatively fast mutation rate and a high copy number per cell, which makes it an ideal marker when using noninvasively collected samples that contain degraded DNA. The Brown region is 935 base pairs long and includes the end of the *ND4* gene, beginning of the *ND5* gene, and the intervening tRNAs (Brown et al. 1982). Cytochrome b (*CYTB*) is a 1,141 base pair long protein-coding region. Both of these loci have been used extensively in studies of Old World Monkey evolutionary genetics and have been shown to provide an accurate account of female population history (e.g., Brown et al. 1982; Telfer et al. 2003; Newman et al. 2004; Wildman et al. 2004; Ting 2008; Zinner et al. 2009).

### DNA Extraction, PCR amplification, and DNA sequencing

Total genomic DNA was extracted from fecal samples using the Qiagen DNA stool kit (Qiagen catolog number 51504) with modified protocols (overnight incubation in lysis buffer, half InhibitEX tablet, additional proteinase K, reduced elution volume) in the labs of N. Ting and C. Roos. DNA was extracted from tissue biopsies through a phenol chloroform extraction in the lab of N. Phillips soon after collection in 2007, stored at −20°C, then processed through a whole-genome amplification in 2009 (Illustra GenomiPhi HY DNA Amplification Kit; GE Healthcare Life Sciences). Amplification primers, sequencing primers, and cycling parameters for the Brown region and *CYTB* were from Newman et al. (2004) and Zinner et al. (2009), respectively. To ensure that these primers would not amplify nuclear pseudogenes in the drill, we compared Brown and *CYTB* sequences from one Bioko tissue sample that were sequenced from two different amplification protocols: (1) standard PCR and (2) a whole mitochondrial genome amplification via long-range PCR. This mitochondrial genome was amplified in two 10,000 base pair overlapping halves, and the overlapping reads were sequenced and compared to ensure a circular molecule of mitochondrial origin (following the methods of Thalmann et al. 2004; Raaum et al. 2005; primers available upon request). The Brown and *CYTB* sequences from the two different amplification protocols were identical, demonstrating that the standard short-range PCR primers are unlikely to amplify nuclear DNA. We also visually confirmed the coding frames and translated the protein-coding regions in our dataset in MacClade v4.08 (Maddison and Maddison 2005) to check for unexpected stop codons, and we found no evidence that our primers amplified nuclear pseudogenes. PCR products were cleaned using exonuclease I and shrimp alkaline phosphatase (Hanke and Wink 1994), cycle sequencing was performed using the Big Dye kit (Big Dye v3.1) following the manufacturer's protocol for diluted reactions, and products were run on an ABI PRISM 3130xL or 3730 DNA Sequencer. Complementary strands were sequenced from multiple PCR products to ensure the fidelity of the data, and the sequences were edited and assembled using Geneious v5.1.3 (Drummond et al. 2010). These data have been deposited in GenBank under accession numbers found in Table S1. Alignments of the Brown region, *CYTB*, and a combined dataset were constructed using default values in ClustalW v2.0 (Larkin et al. 2007).

### Population structure and relationship analyses

The presence of population structure can be mistaken for a genetic signature of population size decline. This is because many methods used to infer changes in past population size use models (e.g., Wright–Fisher, Moran) that do not account for structured populations. However, this is most problematic when there are cases of intermediate gene flow and recent immigration between subpopulations (Peter et al. 2010). In species with subpopulations that share very little gene flow, alleles coalesce in each subpopulation and a nonstructured population model may apply again (Chikhi et al. 2010). We expect this latter circumstance to apply to this study because we are using a matrilineal marker in a species that is likely female philopatric. With little movement of females between subpopulations, there should be a high amount of population structure among drill mitochondrial lineages. In order to assess this possibility, we used Arlequin v3.5 (Excoffier and Lischer 2010) to calculate *F*_ST_ values among the Nigerian, KNP, Ebo Forest, and Bioko Island subpopulations using the combined dataset, conventional *F*-statistics, and 1000 permutations. In order to display the distribution of variation within the species, we constructed a haplotype network using statistical parsimony (Templeton et al. 1992) in the R package PEGAS (Paradis 2010). This method of network construction has been shown to have lower error rates than many other network construction methods (Woolley et al. 2008). We also inferred a Bayesian gene tree and coalescent dates using the BEAST v1.6.1 package (Drummond and Rambaut 2007). For this analysis, we conservatively discarded the first 25% of trees from two BSP runs (see below) and combined them using LogCombiner. We then processed the tree file in TreeAnnotator and visualized the tree in FigTree v1.3.1.

### Evolutionary rate analysis

We obtained separate Brown region and *CYTB* rates of evolution for use in the BSP analysis (see below) using both newly sequenced data and available nucleotide data in GenBank from genera in the tribe Papionini. Brown and *CYTB* datasets were generated by aligning orthologous sequences in *Papio hamadryas* (Y18001), *Theropithecus gelada* (FJ785426), *Lophocebus albigena* (JQ068156–Brown, JQ068153–*CYTB*), *Cercocebus torquatus* (JQ068155–Brown, JQ068152–*CYTB*), *M. sphinx* (JQ068154–Brown, JQ068151–*CYTB*), and *M. leucophaeus* (JQ068213–Brown, JQ068159–*CYTB*) using default parameters in the program ClustalW v2.0 (Larkin et al. 2007). The *Lophocebus, Cercocebus,* and *Mandrillus* sequences were previously unpublished data collected using the methods of Raaum et al. (2005). Modeltest 3.8 (Posada and Crandall 1998) and PAUP* v4b10 (Swofford 2003) were used to determine that the Hasegawa, Kishino, and Yano (HKY; Hasegawa et al. 1985) model was the best fit for both Brown and *CYTB* data under the Akaike Information Criterion (AIC; Akaike 1974), with the former under a Gamma distribution (HKY + G) and the latter with a proportion of invariant sites (HKY + I) (Yang 1994). We used the BEAST v1.6.1 (Drummond and Rambaut 2007) package to estimate rates under an exponentially distributed relaxed uncorrelated clock model and a Yule speciation process tree model. Fossil data were used to set the divergence of *Papio* and *Theropithecus* to 5.0 ± 0.5 million years ago under a normal distribution (95% CI: 6–4 million years ago; Leakey 1993; Delson 2000). Four categories of the Gamma distribution were used for the Brown region, and all other priors for model parameters and statistics were left at default values for both datasets. For each alignment, two 10,000,000 generation runs were conducted with parameters logged every 1000 generations, and log files were combined in LogCombiner after conservatively discarding the first 25% of samples as burn-in. Tracer v1.5 (Rambaut and Drummond 2009) was used to assess chain convergence and view the combined logfiles for both alignments, which yielded indistinguishable rates of evolution when rounded to the nearest 10^7^ (3 × 10^–7^ sub/site/generation; 9 × 10^–8^ lower 95% Highest Posterior Density; 5 × 10^–7^ upper 95% Highest Posterior Density). Although female generation time (average age of reproduction; Kimura and Crow 1963) in wild drills is largely unknown, we estimate it to be 10–12 years. This number is derived using captive life history data from the Pandrillus colony in Nigeria (Wood 2007) on mean female age at first offspring birth (4.5 years), mean interbirth interval (1.4 years), and estimated lifespan (no more than 20 years, the age at which captive individuals deteriorate considerably; ELG personal observation). The average age at first birth and interbirth interval are similar to those from the semifree ranging mandrill (*M. sphinx*) colony at Lope (Setchell et al. 2001, 2002, 2005), and a 12-year generation time is consistent with that which has been used in the closely related and similarly sized yellow baboon (Rogers and Kidd 1993).

### Effective population size change analyses

We used DnaSP 5.0 (Librado and Rozas 2009) to calculate basic summary statistics on the combined Brown region and *CYTB* dataset. In order to assess past changes in female effective population size (*N*_ef_), we calculated Fu's *F_s_* (Fu 1997) and *R*_2_ (Ramos–Onsins and Rozas 2002). For both tests, we generated null distributions from 10,000 coalescent simulations of a constant-sized population to assess the significance of our results. We also used the BEAST 1.6.1 package to analyze the drill Brown region and *CYTB* alignments through a BSP, which infers the timing and magnitude of past changes in population size (see Supporting Information for alignments and the XML file). Because site and clock models were found to be the same in the within species alignments for both regions, we analyzed the two datasets under the following linked substitution, clock, and tree models. The HKY model was found to fit both datasets best according to the AIC in Modeltest 3.8, and the clock model was set to a strict clock (because we believe rate heterogeneity in mitochondrial protein-coding regions within a species such as the drill to be unlikely) with a 3.0 × 10^–7^ sub/site/generation rate of evolution (12-year generation time). We also ran the analysis using a rate of 2.5 × 10^–7^ sub/site/generation, which is the evolutionary rate if generation time is 10 years and average female drill lifespan is approximately 16 years. We used a Bayesian Skyline coalescent tree prior with 10 groups under a piecewise-constant model. Priors for model parameters and statistics were left at default values. The analysis was run for 20 million generations with parameters logged every 1000 generations, and Tracer 1.5 was used to inspect chain convergence and conduct the skyline reconstruction. We also ran the analysis twice to make sure results were consistent between runs and chains had converged. To assess whether the BSP generated the demographic scenario that best fits the data, we repeated this analysis under a constant population size tree model. A Bayes Factor Test (Kass and Raftery 1995) was then conducted in Tracer 1.5 on the likelihood traces to compare the fits of the BSP and constant population size models to the data (Suchard et al. 2001).

We also conducted BSP analyses on numerous resampled datasets to determine the effects of unequal samples sizes among the four different populations (KNP *n*= 33, Nigeria *n*= 6, Ebo Forest *n*= 5, Bioko Island *n*= 10), assess potential population size changes in each population, and to investigate the effect of removing certain populations. The resampled datasets included KNP, Nigerian, and Ebo Forest populations individually (Bioko Island could not be analyzed alone using a BSP because it contained only one haplotype), Nigerian and Ebo Forest populations individually but randomly resampled to each containing 35 sequences, all populations but with the KNP population randomly resampled to 10 sequences, a KNP-Nigeria-Ebo Forest dataset (Bioko excluded), and a KNP-Nigeria dataset (Bioko and Ebo Forest excluded). All priors in the resampling analyses were kept consistent with the initially described BSP analysis. In addition, we repeated the original BSP analysis with an empty alignment to test the influence of the priors on the demographic results.

## Results

**Figure 4:**
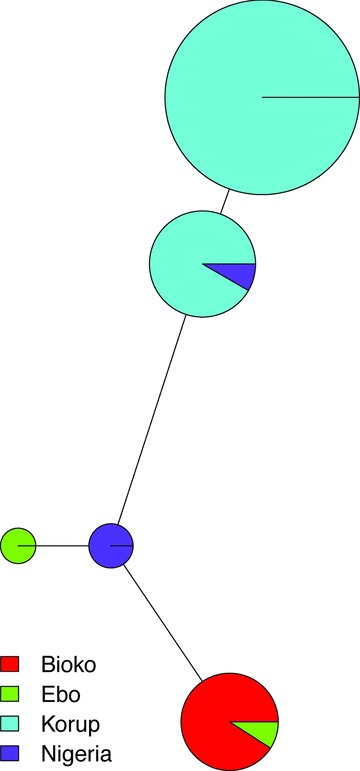
Statistical parsimony network of *Mandrillus leucophaeus* mitochondrial haplotypes. Five haplotypes are present in the sampled populations. Size of circle is proportional to frequency of each haplotype. Very little variation is shared between the different sampled populations.

**Figure 5:**
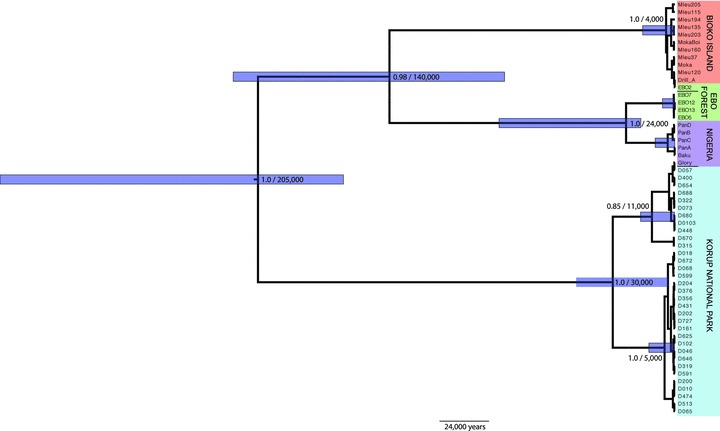
Bayesian gene tree of *Mandrillus leucophaeus* mitochondrial lineages. Tree was inferred using a Bayesian Skyline Plot (BSP) tree prior in the BEAST v1.6.1 package (Drummond and Rambaut 2007). Shown at major nodes are posterior probabilities, coalescent times (years, rounded to the thousand), and 95% confidence intervals. With the exception of two individuals (EBO2, Glory), all individuals group with other members from their respective localities.

*F*_ST_ values were all above 0.5 (*P* < 0.05; Table 1), indicating that drill mitochondrial lineages are highly structured between Nigeria, KNP, Ebo Forest, and Bioko Island. The haplotype network (Fig. 4) shows one haplotype exclusively found in KNP, one haplotype shared between KNP and Nigeria, a haplotype found exclusively in Nigeria that is closely related to a haplotype found exclusively in Ebo Forest, and a haplotype shared between Ebo Forest and Bioko Island. The gene tree (Fig. 5) shows close relationships between Ebo Forest and Bioko Island, and those two localities and Nigerian localities. All modern drill mitochondrial lineages coalesce to a date of 205,000 years ago (95% CIs: 162,500–331,000).

**Table 1:** Pairwise *F*_ST_ values among sampled drill (*Mandrillus leucophaeus*) subpopulations computed using conventional *F* -statistics from haplotype frequencies (*P* < 0.05).

	Bioko	Ebo	Nigeria	Korup
Bioko	—			
Ebo	0.845	—		
Nigeria	0.884	0.636	—	
Korup	0.684	0.556	0.550	—

**Figure 6:**
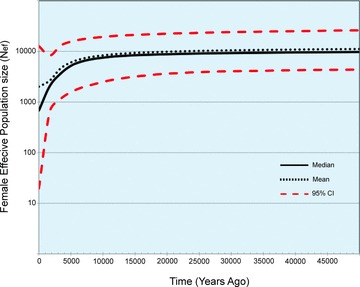
Bayesian Skyline Plot (BSP) displaying changes in female effective population size (*N*_ef_) through time in *Mandrillus leucophaeus* based on 2,076 base pairs of mitochondrial DNA. Present day is on the left on the *x* -axis. A decline in *M. leucophaeus N*_ef_ begins near the onset of the Last Glacial Maximum (LGM) and becomes most severe during the Mid Holocene (5Ka).

Values of Fu's *F_s_* and *R*_2_ were significantly different than expected under a model of constant population size (Table 2), with the observed values falling outside the 95% confidence intervals of the simulated values. The BSP indicates a nearly 15-fold decrease in *N*_ef_ that starts at the end of the Pleistocene and continues throughout the Holocene (Fig. 6). Specifically, median *N*_ef_ changes from 9,950 (95% CI = 4,112–28,777) to 681 (95% CI = 20–12,658). The decrease starts gradually at the beginning of the LGM (∼20–25 Ka), continues to the end of the Pleistocene and beginning of the Holocene, and becomes particularly sharp and pronounced in the Mid Holocene around 5 Ka. Between 2 and 1 Ka, the 95% CI becomes much larger and the mean *N*_ef_ levels off slightly, although it still shows a declining trend. The Bayes Factor Test provides strong support for the Bayesian Skyline model when compared to a model of constant population size (log_10_ Bayes Factor = 3.131). The BSP analysis that used a shorter generation time showed a very similar result (Fig. S1). The resampled datasets all showed declines in *N*_ef_ to varying degrees except for the analysis of the KNP only dataset, whose mitochondrial lineages only allowed demographic inference of 10 generations into the past (Fig. S2). The analysis performed with the described priors and no data also resulted in a skyline with no change in *N*_ef_.

**Table 2:** Summary statistics and results of tests for population size change as calculated in DnaSP 5.0 for the drill (*Mandrillus leucophaeus*).

*N*_seq_	*n*_sites_	S	Cs	Cn	*h*	Hd	π	π_s_	k	*F*_s_	*R*_2_
54	2076	39	32	6	5	0.743	0.00689	0.02586	14.3	22.124^*^	0.1834^*^

*N_seq_*= total number of sequences analyzed; *n_sites_*= total number of sites analyzed; S = number of polymorphic (segregating) sites; Cs = number of synonymous polymorphic sites; Cn = number of nonsynonymous polymorphic sites; *h*= number of haplotypes; *Hd*= haplotype diversity; π= nucleotide diversity; π_s_= silent nucleotide diversity; k = average number of nucleotide differences; *F_s_*= Fu's *F_s_*; *R*_2_= Ramos-Onsins and Rozas *R_2_* statistic; *=*F_s_* and *R*_2_ results differed significantly from expectations based on the null model of constant population size.

## Discussion

### Robustness of demographic analysis

Our BSP analysis suggests a nearly 15-fold decrease in drill female effective population size (*N*_ef_) that began in the Late Pleistocene. We believe this demographic result to be statistically robust. The results of the *F_s_* and *R*_2_ tests suggest that drill female effective population size (*N*_ef_) may not have been constant in the past, and the results of the Bayes Factor Test demonstrates the BSP model was a better fit to the data than a model of constant population size. We also performed the BEAST analysis using the described priors with no data and recovered a flat skyline plot, thus indicating that the priors did not contribute to the inferred demographic history.

While population structure can often be mistaken for a decline in population size, we do not believe this to be the case here. Signals of population structure and population decline are most commonly confused when there are intermediate levels of gene flow and divergent alleles are introduced from an unsampled subpopulation, thus mimicking the patterns of genetic diversity one expects from a population decline. To specifically avoid this, we employed a sampling strategy that incorporated the probable major subpopulations across the drill range (Chikhi et al. 2010). Furthermore, this issue is not as problematic when the population is extremely structured with little gene flow among demes (Chikhi et al. 2010). The very high *F*_ST_ values among the drill subpopulations indicates this is the case in this dataset, as would be expected when using a matrilineal maker in a female philopatric species. In such a circumstance, it is likely that alleles coalesce in each subpopulation and the inferred demographic collapse occurred across most demes. Our resampling analyses show that this was likely the case. Almost all resampled datasets resulted in a BSP that showed declines in *N*_ef_ since the Late Pleistocene, with more severe declines occurring when sample sizes were higher. Removing certain populations or equalizing sample sizes among populations had little effect, and individual analyses of the Nigerian and Ebo Forest populations showed population declines regardless of sample size. The lone exception is the KNP population, which failed to show a population size change. However, this result is not surprising because the mitochondrial lineages in this population had a recent coalescent time and only the past 120 years of demographic history were recovered in the BSP.

### Demographic history of the drill

Because the study animal exists in a region with an exceptional palynological record, past changes in *N*_e_ can be directly correlated with known environmental change. Our hypothesis was that the drill would have experienced changes in population size that mirror changes in forest coverage. Our BSP analysis indeed suggests such a trend; a gradual decrease in drill *N*_ef_ that began prior to the LGM when global temperatures were cooling, and the decrease persisted throughout the LGM when forests in West Central Africa saw a dramatic reduction. However, during the period of forest expansion following the LGM (10–3 Ka), we do not see evidence of a population size increase. *N*_ef_ continues to decline after the LGM and through the Early Holocene, and it abruptly declines in the Middle Holocene. There is, however, a slight leveling off of this decline in mean *N*_ef_ between 2 and 1 Ka when the 95% CIs also become much larger. This last stage of the BSP with large margins for error may represent the limits of the mitochondrial data, where recent demographic events are difficult to detect.

### Causes of inferred population collapse

Although changes to the environment can be a major influence on population size dynamics, both competition and disease are known to affect animal abundance as well (Chapman et al. 1999). However, we believe interspecific competition was likely not a primary factor here because both the drill's large body size and social group size would provide significant advantages in competition for resources. While intraspecific competition (resulting from resource depletion) or disease remain possible causes, we believe environmental change induced by changes in climate to be the most likely primary explanation given that the timing of the population collapse coincides with known changes in forest coverage in this area. It should also be noted that these explanations are not mutually exclusive and can have a synergistic effect. For example, environmental disturbance can lead to stress, increased competition, and disease, all of which can act together to affect population dynamics in a species (Chapman et al. 2006).

Given that climate change is the likely primary driver of the inferred demographic history, it is surprising that there was no evidence of a drill population size increase during the period of warm, humid climate and forest expansion in the Early and Middle Holocene. This is counter to refugia theory expectations (Haffer 1982; Mayr and O’Hara 1986), which postulate that animal population range expansions and contractions should mirror climate-induced changes in habitat. This should particularly be the case in the drill, which has a highly diverse and adaptable diet and the ability to use resources in periods of drought that other sympatric primates cannot use, such as hard seeds (Astaras 2009; Astaras and Waltert 2010). A likely explanation is that our data do not provide sufficient resolution to detect a closer correlation between drill *N*_ef_ and forest coverage. Use of a single locus may preclude inference of changes in *N*_e_ that predate the most recent detectable demographic event, especially if that event was particularly severe (Heled and Drummond 2008). This is because a single locus will provide fewer coalescent points from which *N*_e_ can be inferred compared to multiple loci. It is thus likely that the population decline seen in the BSP is primarily related to a reduction in forest coverage that began approximately 3 Ka (Fig. 3; Maley 2002). This general time period on the BSP is when the population collapse becomes most pronounced. Evidence of the collapse beginning earlier could thus be a residual effect of this event, a signature of an earlier demographic collapse related to the LGM, or a general lack of resolution.

Whether or not the initial population decline was caused by the onset of the LGM, and whether or not there was a population expansion in the Early Holocene, it is clear that environmental conditions in the Mid-Late Holocene have had a dramatic effect on drill population dynamics and modern drill genetic diversity. Although some studies identified recent (i.e., the past few hundred years) habitat changes by humans as the main source of past population size change and current genetic variation in tropical mammals (Goossens et al. 2006; Olivieri et al. 2008; Craul et al. 2009; Thalmann et al. 2011), other studies are consistent with our conclusion that Mid-Late Holocene events were particularly influential (Heller et al. 2008; Okello et al. 2008; Lawler 2011). This time period saw an increase in aridity and the opening up of forests across Africa. Although this fragmentation facilitated the westward movement of Bantu-speaking populations into Central and West Africa, it is unlikely that human activities are responsible for the forest-cover changes reported for this area at the time (Maley 2002). Our results thus demonstrate the effects that climate change had on drill population dynamics around 3 Ka.

### Implications for future climate change

Modern increases in global temperature are already having an effect on wildlife communities (Hughes 2000; Walther et al. 2002; Walther 2010), but predicting long-term responses to current climate change requires data from a multitude of sources. Investigating the effects of past climate change is thus one way of understanding how future climate change might affect the population dynamics and distributions of natural communities (Cordellier and Pfenninger 2009; Prost et al. 2010). The climate of the Mid-Late Holocene was characterized by an increase in aridity and seasonality during a warm interglacial period; and this is potentially analogous to current conditions where tropical climates are projected to experience increases in both temperature and aridity (Maley 2002). This is due to global warming and human-mediated forest loss that disrupts the moisture cycle and may lead to increased drought and seasonality (Hannah et al. 2007; Hannah and Lovejoy 2007; Heubes et al. 2011). Based on the findings of this study, forest-adapted species such as the drill may not be able to cope with such changes and their populations could dramatically decrease—a catastrophic scenario for a species already on the brink of extinction. Limiting forest loss by protecting large tracts of intact tropical forest will therefore pay dividends beyond just the protection of wildlife habitat from poaching and human development—it will aid in maintaining the evapotranspiration cycle and potentially prevent an increase in aridity and seasonality that could have negative effects on species such as the drill.

## Conclusions

We conducted a study of changes in past effective population size since the LGM in a tropical rainforest-adapted mammal to see how it responded to periods of past climate change. Our study animal was the drill, which is a poorly known and endangered primate (Oates 1996), and this research is the first genetic work to be conducted on this species. We predicted that drill population dynamics would closely mirror changes in West Central African forest coverage since the Late Pleistocene. Our data suggest that the drill underwent a population collapse that was most severe during the Mid Holocene, which supports the conclusions of other studies of sub-Saharan mammals that found events during this period to have had a dramatic impact on population dynamics and modern genetic diversity (Heller et al. 2008; Okello et al. 2008). In West Central Africa, this coincides with a reduction of forest coverage associated with warm temperatures and increased aridity and seasonality, and is potentially analogous to the foreseen global warming effects in the region. In order to prevent or at least curb future population reductions in rainforest-adapted species such as the drill, large areas of forest must be protected to both preserve natural habitat and prevent disruptions in the moisture cycle. Future research that incorporates more genetic loci and multiple sympatric taxa will provide better resolution in reconstructions of demographic history and shed further light on how species might respond to the same ecological changes. Such information is essential for the better design of landscape scale—rather than species focused—conservation strategies in the region.

## Supporting Information

**Figure S1.** Bayesian Skyline Plot displaying changes infemale effective population size (N_ef_) through time in*Mandrillus leucophaeus* based on 2,076 base pairs ofmitochondrial DNA, a 10 year generation time, and a 2.5 ×10^−7^ sub/site/generation mutation rate. Presentday is on the left on the x-axis. A decline in *M.leucophaeus* N_ef_ begins near the onset of the Last Glacial Maximum and becomes most severe during the Mid Holocene (5Ka).

**Figure S2.** Bayesian skyline plots of resampled data. Shown are the median and 95&percnt; confidence intervals. Time is on x-axis with units in generations and a maximum value of 5000, except for d) which has a maximum value of 20. Female effective population size on y-axis with a maximum value of 100,000 individuals. a) All populations with Korup National Park (KNP) population resampled to 10 individuals. b) KNP, Nigeria, and Ebo Forest populations (Bioko Island excluded). c) KNP and Nigeria populations (Ebo Forest and Bioko Island excluded). d) KNP population only (generation time on x-axis with a maximum value of 20). e) Ebo Forest population only. f) Ebo Forest population resampled to 35 individuals. g) Nigeria population only. h) Nigeria population resampled to 35 individuals.

**Table S1.** Sampled individuals with associated localitydata and Genbank accession numbers. Biomaterials from Biokoindividuals with an “Mleu&num;” ID were fingertips (115, 120, 135, 160, 37) or liver (194, 203, 205) from fresh bushmeat carcasses. All other biomaterials were of fecal origin.
