# Supplementary figures and images for: Genetic signatures of a demographic collapse in a large-bodied forest dwelling primate (Mandrillus leucophaeus)
Source: Ecol Evol. 2012 Mar;2(3):550–61. doi: 10.1002/ece3.98 (PMC3399144; doi:10.1002/ece3.98)

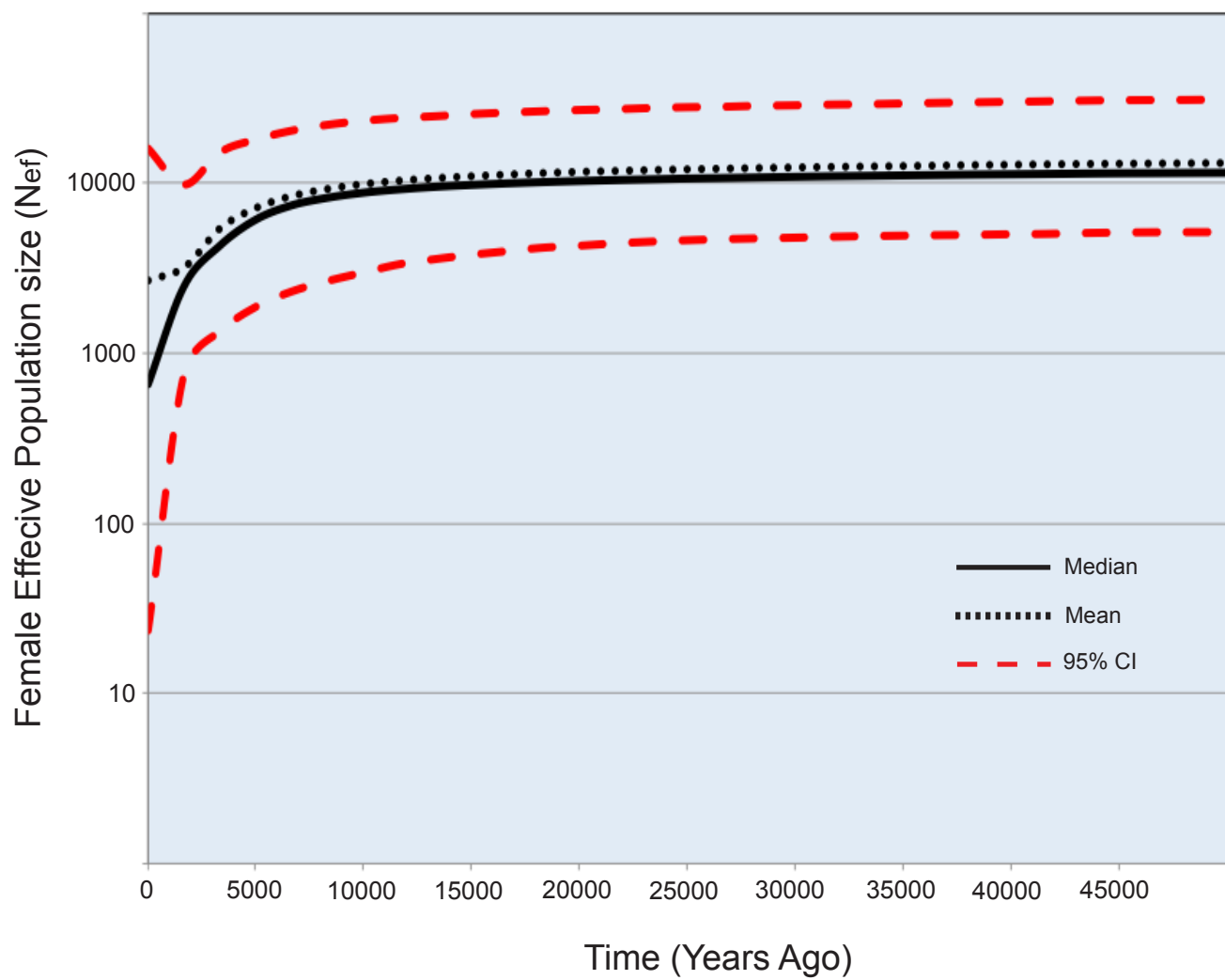

Supplement: Supplementary file 1 [file ece30002-0550-SD1.pdf]

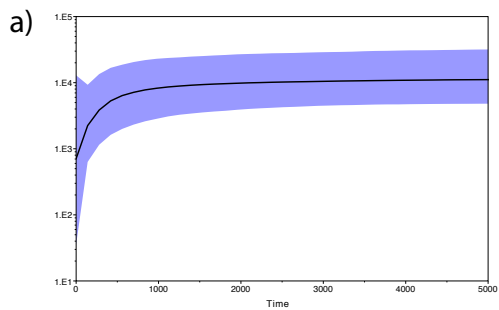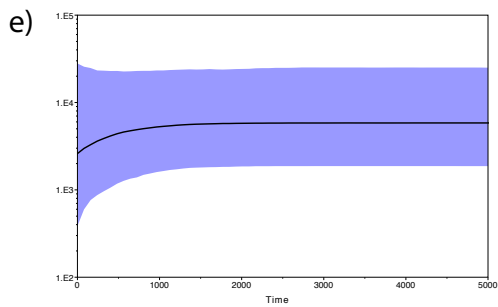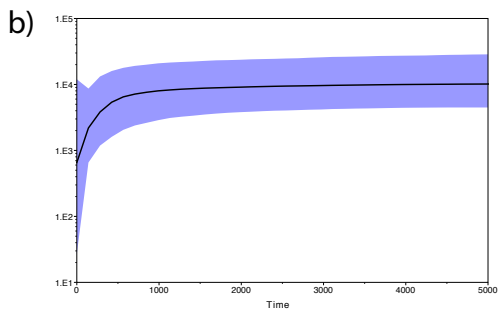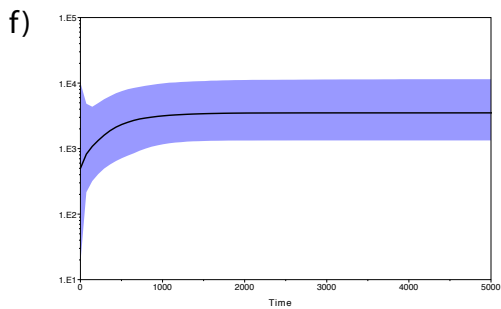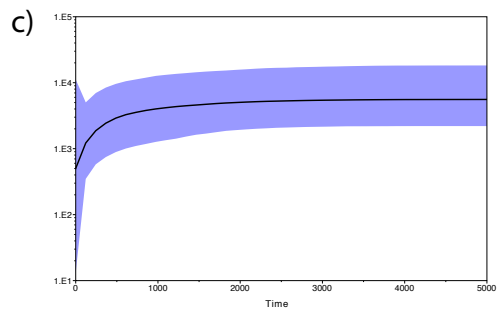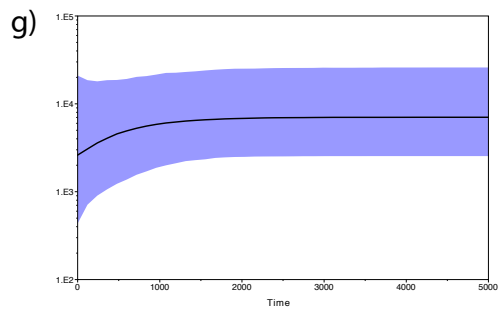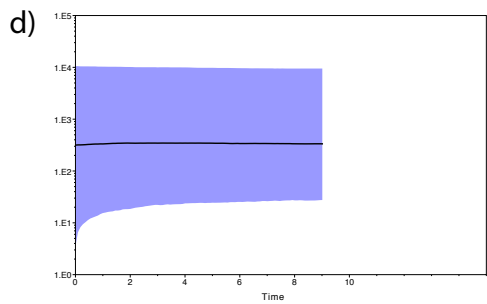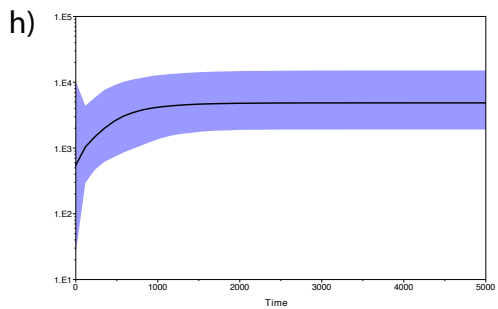

Supplement: Supplementary file 2 [file ece30002-0550-SD2.pdf]
