# Supplementary material for: Genetic signatures of a demographic collapse in a large-bodied forest dwelling primate (Mandrillus leucophaeus)
Source: Ecol Evol. 2012 Mar;2(3):550–61. doi: 10.1002/ece3.98 (PMC3399144; doi:10.1002/ece3.98)
Supplement: Supplementary file 3 [file ece30002-0550-SD3.docx]

**Table S1**: Sampled individuals with associated locality data and Genbank accession numbers. Biomaterials from Bioko individuals with an “Mleu#” ID were fingertips (115, 120, 135, 160, 37) or liver (194, 203, 205) from fresh bushmeat carcasses. All other biomaterials were of fecal origin.

**Genbank#**

**ID Origin/Locality Brown *CYTB***

Baku Northern Cross River (i.e., Okwangwo and Afi), Nigeria JQ068222 JQ068168

Glory Southern Cross River (i.e., Western Oban Hills), Nigeria JQ068242 JQ068188

PanA Northern or Southern Cross River, Nigeria JQ068223 JQ068169

PanB Northern or Southern Cross River, Nigeria JQ068224 JQ068170

PanC Northern or Southern Cross River, Nigeria JQ068225 JQ068171

PanD Northern or Southern Cross River, Nigeria JQ068226 JQ068172

Mleu115 Bioko Island, Equatorial Guinea JQ068211 JQ068157

Mleu120 Bioko Island, Equatorial Guinea JQ068215 JQ068161

Mleu135 Bioko Island, Equatorial Guinea JQ068220 JQ068166

Mleu160 Bioko Island, Equatorial Guinea JQ068212 JQ068158

Mleu194 Bioko Island, Equatorial Guinea JQ068216 JQ068162

Mleu203 Bioko Island, Equatorial Guinea JQ068213 JQ068159

Mleu205 Bioko Island, Equatorial Guinea JQ068217 JQ068163

Mleu37 Bioko Island, Equatorial Guinea JQ068218 JQ068164

Drill_A Bioko Island, Equatorial Guinea JQ068214 JQ068160

Moka Bioko Island, Equatorial Guinea JQ068221 JQ068167

EBO12 Ebo Forest, Cameroon JQ068227  JQ068173

EBO13 Ebo Forest, Cameroon JQ068228  JQ068174

EBO5 Ebo Forest, Cameroon JQ068229 JQ068175

EBO7 Ebo Forest, Cameroon JQ068230 JQ068176

EBO2 Ebo Forest, Cameroon JQ068219 JQ068165

D046 Korup National Park, Cameroon JQ068239 JQ068185

D065 Korup National Park, Cameroon JQ068238 JQ068184

D068 Korup National Park, Cameroon JQ068237 JQ068183

D200 Korup National Park, Cameroon JQ068236 JQ068182

D431 Korup National Park, Cameroon JQ068235 JQ068181

D474 Korup National Park, Cameroon JQ068234 JQ068180

D599 Korup National Park, Cameroon JQ068233 JQ068179

D625 Korup National Park, Cameroon JQ068231 JQ068177

D646 Korup National Park, Cameroon JQ068232 JQ068178

D057 Korup National Park, Cameroon JQ068240 JQ068186

D073 Korup National Park, Cameroon JQ068241 JQ068187

D010 Korup National Park, Cameroon JQ068243 JQ068189

D018 Korup National Park, Cameroon JQ068244 JQ068190

D102 Korup National Park, Cameroon JQ068245 JQ068191

D161 Korup National Park, Cameroon JQ068246 JQ068192

D202 Korup National Park, Cameroon JQ068247 JQ068193

D204 Korup National Park, Cameroon JQ068248 JQ068194

D319 Korup National Park, Cameroon JQ068249 JQ068195

D356 Korup National Park, Cameroon JQ068250 JQ068196

D376 Korup National Park, Cameroon JQ068251 JQ068197

D513 Korup National Park, Cameroon JQ068252 JQ068198

D591 Korup National Park, Cameroon JQ068253 JQ068199

D672 Korup National Park, Cameroon JQ068254 JQ068200

D727 Korup National Park, Cameroon JQ068255 JQ068201

D0103 Korup National Park, Cameroon JQ068256   JQ068202

D315 Korup National Park, Cameroon JQ068257 JQ068203

D322 Korup National Park, Cameroon JQ068258 JQ068204

D400 Korup National Park, Cameroon JQ068259 JQ068205

D448 Korup National Park, Cameroon JQ068260 JQ068206

D654 Korup National Park, Cameroon JQ068261 JQ068207

D670 Korup National Park, Cameroon JQ068262 JQ068208

D680 Korup National Park, Cameroon JQ068263 JQ068209

D688 Korup National Park, Cameroon JQ068264 JQ068210
